# Impact of a Community-Based Health Intervention on Health Knowledge, Lifestyle Goals, Dietary Practices, and Physical Activity in Older Adults: A Multisite Cohort Study

**DOI:** 10.3390/healthcare12242588

**Published:** 2024-12-23

**Authors:** Shanice Wei En Lim, Jesslyn Hwei Sing Chong, Jia Yi Chee, Charles Chin Han Lew, Hee Hoon Lee, Lancelot Kher Wi Chua, Kian Chong Lim, Hui An Koh, Bao Yin Sow, Janelle Wood, Dorcas Gui, Adrian Ujin Yap

**Affiliations:** 1 Ng Teng Fong General Hospital, National University Health System, Singapore 609606, Singapore; lim_wei_en_shanice@nuhs.edu.sg (S.W.E.L.); sow_bao_yin@nuhs.edu.sg (B.Y.S.); janelle_wood@nuhs.edu.sg (J.W.);; 2 Saw Swee Hock School of Public Health, National University of Singapore, Singapore 117549, Singapore; e0802485@u.nus.edu; 3 Faculty of Health and Social Sciences, Singapore Institute of Technology, Singapore 138683, Singapore; 4 Faculty of Dentistry, National University Health System, Singapore 119228, Singapore; 5 Duke-NUS Medical School, Singapore Health Services, Singapore 169857, Singapore

**Keywords:** community-based health intervention, preventive health program, diet, physical activity, health knowledge, older adults, goal setting

## Abstract

**Background:** Studies on the effectiveness of community-based health interventions (CBHIs) in improving lifestyle practices among older adults are limited. **Objectives:** This study evaluated the impact of a CBHI on health knowledge, lifestyle goal attainment, and practices among older adults in Singapore. **Methods:** Older adults (aged ≥60 years) were recruited from 29 senior recreation centres to participate in the “Wise and Well” programme, a 3-month CBHI designed to empower individuals to make healthier lifestyle choices. Pre- and post-programme assessments of health knowledge, goal attainment, and lifestyle behaviours (dietary practices and physical activity levels) were conducted using a health knowledge questionnaire, Goal Attainment Scaling (GAS), the Modified Dietary Practice Questionnaire (DPQ), and the Global Physical Activity Questionnaire (GPAQ), respectively. Statistical analysis was performed using the Wilcoxon signed-rank and McNemar’s tests (α = 0.05). **Results:** The study comprised 464 participants (mean age 73.1 years [SD 7.6]; 75.9% women). Three months post-program, participants showed significantly increased health knowledge (*p* < 0.001) and improved lifestyle goal attainment (*p* < 0.001). Additionally, there was a significant increase in the proportion of participants meeting or exceeding fruit (*p* < 0.001) and vegetable (*p* = 0.002) intake recommendations and reductions in sugar-sweetened beverage consumption (*p* = 0.002). However, no significant changes were observed in fried food consumption or physical activity levels. High health knowledge scores were associated with 2.17 (95% confidence interval 1.02,4.64) times greater odds of increased vegetable intake. **Conclusions**: The CBHI shows potential as an effective approach to improving health knowledge and enhancing lifestyle goals and practices among older adults

## 1. Introduction

The global population is ageing rapidly, with the number of individuals aged 60 and above projected to more than double to 2.1 billion by 2050 [1]. Likewise, Singapore—a small, developed, and densely populated country in Southeast Asia—is experiencing one of the fastest rates of population ageing. Estimates suggest that by 2030, Singapore will be classified as a “*super-aged society*”, with 1 in 4 Singaporeans aged 65 years or older [2,3]. As the population ages, the prevalence of non-communicable diseases (NCDs) and age-related frailty is expected to rise exponentially [4]—placing an increasing strain on healthcare systems and society as a whole. This underscores the urgent need for innovative and targeted strategies to alleviate this burden by addressing modifiable lifestyle-related risk factors, including poor dietary practices and physical inactivity, among older adults [5,6,7].

Community-based health interventions (CBHIs) may help to reduce the chronic disease burden by targeting modifiable lifestyle risk factors, particularly poor dietary practices and physical inactivity [8]. These programmmes leverage social networks and environmental contexts to drive behaviour change through group education, peer support, and community engagement [9]. Directly transferring these interventions to Asian contexts—such as Singapore’s multicultural, multilingual, and high-density urban environment—may be challenging. Sociocultural differences, including dietary norms centred around hawker cuisines and social structures that influence caregiving, can influence the effectiveness and acceptability of interventions. Health literacy levels and engagement patterns also vary widely, necessitating culturally sensitive adaptations beyond a simple translation of Western models. A handful of CBHIs for older adults exist locally, including chronic disease self-management workshops and active ageing initiatives [10,11,12]; however, these interventions [13,14,15,16] are often narrowly focused, lack scalability, and may not fully resonate with the diverse preferences, resources, and literacy levels of older adults in Singapore.

Complicating matters further, the uptake of preventive health interventions among older adults in Singapore remains suboptimal. Low health literacy can impede their understanding of self-care practices and reduce their willingness to adopt healthier habits [10,11]. Meanwhile, practical barriers—such as mobility limitations in navigating the urban environment, financial constraints in selecting healthier food options, and social isolation that diminishes ongoing support—add complexity to designing interventions that are both effective and flexible [17,18].

Against this backdrop, a more personalised and adaptive approach is needed. Integrating a standardised, measurable goal-setting tool within a CBHI offers a viable solution to address these challenges, enabling older adults to identify personalised health goals that enhance self-efficacy and foster sustained engagement [19]. Research suggests that combining goal setting with CBHI strategies significantly improves participant engagement and health outcomes, particularly regarding modifications in dietary practices and physical activity [20,21]. Hence, integrating a standardised structured and measurable goal-setting assessment tool into group-based CBHIs offers a promising solution to these limitations by providing clear and personalised pathways for behaviour change while enhancing accountability, motivation, and adherence over time. Unlike conventional CBHIs that provide uniform recommendations, a goal-setting framework enables participants to define individualised, culturally relevant goals, receive continuous feedback, and gradually adjust targets as their capabilities and circumstances evolve. For instance, older adults with financial constraints can set cost-conscious dietary objectives, while those with mobility issues can prioritise accessible, low-impact exercises. Through this process, goal setting can improve self-efficacy and bridge gaps in health literacy by breaking down complex health behaviours into manageable, clearly communicated steps. These principles align with established health behaviour change theories, such as the Social Cognitive Theory and the Transtheoretical Model, which emphasise the importance of self-efficacy, incremental progress, and tailoring interventions to the individual’s stage of readiness [22,23]. Notably, no existing CBHI in Singapore has incorporated a structured goal-setting framework designed to target this population.

Therefore, this study aims to address the aforementioned knowledge gaps by piloting a multisite cohort study to evaluate the impact of a CBHI incorporating a structured, measurable goal-setting component. Beyond testing its efficacy in this local setting, the study’s findings may inform the adaptation of similar interventions in other rapidly ageing Asian societies. Specifically, it will assess whether integrating a structured goal-setting component can effectively enhance dietary practices and physical activity outcomes. The research hypotheses are as follows:

1. The CBHI improves health knowledge among participants.
2. The integration of structured and measurable goal-setting components within the CBHI enhances lifestyle goal attainment, dietary practices, and physical activity levels.
3. Improvements in health knowledge are associated with better dietary practices and increased physical activity levels.

## 2. Materials and Methods

This study was evaluated by the National Healthcare Group Domain Specific Review Board and was granted exemption from ethical approval requirements due to the use of anonymised retrospective data (reference number: 2025/00120).

### 2.1. Participant Recruitment and Study Design

The study utilised a pre-and post-intervention design. Anonymous data were gathered from participants enrolled in the “Wise and Well” programme initiated by Ng Teng Fong General Hospital (NTFGH). The inclusion criteria included community-dwelling adults aged 60 years or older, Singaporean or permanent residents, and could comprehend Mandarin or English. Interested participants were screened by administrative staff and required to disclose any formal health diagnoses, including physical or psychological disorders or cognitive impairment, as such diagnoses resulted in exclusion from the programme. Individuals with missing or incomplete surveys were excluded. A post-hoc power analysis was performed (G*Power, Version 3.1.9.3) to verify if the acquired sample size was adequate. A conservative assumption of a small effect size (Cohen’s *d* = 0.2) was made given the unknown impact of the programme on health knowledge. Based on a Wilcoxon signed-rank test with alpha and beta set at 5% each, a sample size of 343 was recommended. Since the study sample exceeded this threshold (*n* = 464), statistical analyses were performed to test the research hypotheses.

The programme team engaged managers from recreational, daycare, and senior activity centres, as well as community partners, community interest group leaders, and religious leaders, to promote the programme using publicity materials such as flyers. These centres are accessible community facilities close to public housing complexes, where the majority of Singaporeans reside. Participants who enrolled in the programme provided consent for the use of their collected data for potential programme evaluation and research initiatives.

### 2.2. The “Wise and Well” Programme

The “Wise and Well” programme was implemented across 29 recreation centres across Western Singapore from April 2018 to March 2020. This multi-component, 3-month intervention integrated evidence-based strategies targeting dietary practices, physical activity engagement, and health-promoting lifestyle modifications delivered across 8 sessions (Table 1). Each session lasted 60 min and was conducted in both English and Mandarin.

The programme’s theoretical foundation was grounded in the Social Cognitive Theory, incorporating structured goal-setting mechanisms to address modifiable lifestyle risk factors and enhance the adoption of healthy lifestyle practices. The intervention framework was operationalised through four key behavioural change strategies: knowledge acquisition, self-assessment, skill development, and goal setting. A multidisciplinary team of allied health professionals (AHPs), including dietitians, physiotherapists, and occupational therapists, led the programme’s development and implementation. The curriculum emphasised skill development through experiential learning and visual pedagogical approaches, designed to enhance self-efficacy among older adults, with content culturally adapted to the Singaporean context. The intervention strategically utilised centres and leaders, which serve as established community hubs for older adults’ social engagement and activities in Singapore. This implementation approach leveraged existing social infrastructure to optimise programme delivery, facilitate health information dissemination, and foster peer support networks for sustained behavioural change.

### 2.3. Study Measures

Outcome measures were taken at baseline and at the end of the 3-month intervention using an interviewer-administered survey, which encompassed the following:

#### 2.3.1. Health Knowledge Questionnaire

Health knowledge acquisition was assessed using a structured instrument developed through a systematic process involving an expert panel comprising 6 AHPs. The instrument underwent content validation by the AHPs to ensure comprehensive coverage of the intervention curriculum domains. The questionnaire comprised 18 items across 6 domains, with scores ranging from 0 to 100 (Supplementary Materials).

#### 2.3.2. Lifestyle Goal Attainment Scaling

Originally developed to assess community mental health programmes, Goal Attainment Scaling (GAS) has since been adopted in fields such as neurology and rehabilitation and is now widely used for programme evaluation in research and clinical settings [24,25,26,27,28]. GAS is particularly well-suited for goal setting with older adults, who often face multiple, complex, and highly personalised health issues that are not adequately addressed by standardised assessment tools [24]. Research in geriatric care settings has demonstrated the construct validity of GAS, its responsiveness to change, and its utility as a practical approach to facilitating patient-centred care [24,29,30,31].

GAS was employed during individual goal setting and evaluation sessions through collaborative interactions between AHPs and participants. Its utility lies in its dual function: facilitating the identification and selection of participant-specific, feasible goals while providing a quantitative framework for measuring intervention outcomes. AHPs determined baseline measurements and constructed a standardised 5-point scale for each goal, comprising scores of −2, −1, 0, +1, and +2. A score of 0 represents the anticipated or targeted level of goal achievement. If the expected outcomes were not achieved, scores of −1 or −2 were assigned, depending on the degree of deviation from the target. Conversely, scores of +1 and +2 indicated that the participant exceeded the goal or achieved the optimal desired outcome. These scores were then converted into a numerical T-score, which is normally distributed with a mean of 50 (if goals are met precisely) and a standard deviation of 10 (reflecting overachievement or underachievement). Participants also self-assigned a weighting for each goal, ranging from 1 to 3, with 3 denoting goals that were extremely important or challenging [32].

#### 2.3.3. Dietary Practices

Dietary practices were assessed using a modified version of the validated Dietary Practice Questionnaire (DPQ), first utilised in Singapore’s 2004 National Nutrition Survey [33]. Questions focused on the frequency and quantity of fruit, vegetable, fried food, and sugar-sweetened beverage (SSB) consumption. To ensure standardised measurement, the data collection protocol included visual reference charts depicting standard serving sizes for fruits and vegetables and examples of local fried food varieties and SSBs. These visual aids were carefully designed to help participants estimate their dietary intake more accurately. While the Singapore Health Promotion Board (HPB) recommends a daily intake of two servings each of fruits and vegetables, no specific guidelines exist for fried foods or SSBs [34]. The modified questionnaire preserved the psychometric properties of the original DPQ while tailoring it to meet the study’s contextual requirements.

#### 2.3.4. Physical Activity 

Self-reported PA levels were assessed using the Global Physical Activity Questionnaire (GPAQ)—a tool developed by the World Health Organisation (WHO) [35] and validated for use among Singaporean adults [36]. The GPAQ measures the frequency, duration, and intensity of PA across the three key domains identified by the WHO: work-related, transportation-related, and leisure-time domains. In particular, the “work-related” domain includes both paid and unpaid activities, such as household chores and caregiving. Physical activity was measured as either single sessions or accumulated bouts lasting at least 10 min throughout the day. According to WHO guidelines, adults should engage in at least 150 min of moderate-intensity PA, 75 min of vigorous-intensity PA, or an equivalent combination per week. This corresponds to a minimum of 600 metabolic equivalent of task (MET) minutes per week [37,38].

### 2.4. Statistical Analysis

Statistical analyses were performed using Stata Statistical Software Release 17 (StataCorp LLC, College Station, TX, USA), with the significance level set at 0.05. Categorical data were summarised as counts and percentages, while continuous data were presented as means with standard deviations (SDs) and medians with interquartile ranges (IQRs). Normality was assessed using the Shapiro–Wilk test. As data were not normally distributed, paired analyses were conducted using either the Wilcoxon signed-rank test or McNemar’s test, as appropriate. The intake of fruits and vegetables, as well as physical activity levels, were presented as percentages in line with the National Nutrition Survey and National Population Health Survey [38,39]. SSBs and fried food consumption were reported as servings per week.

To interpret changes in health knowledge scores, participants were dichotomised into two groups using a split-median technique. Scores ≥ 11.1 were classified as “high improvement”, and scores < 11.1 as “low improvement” in health knowledge. The median was chosen as the cutoff due to the absence of predefined thresholds and the non-normal distribution of the data. Changes in dietary practices were similarly dichotomised. For fruits and vegetables, a change was classified as “no increase” if the daily servings increased by ≤0 or as an “increase” if servings increased by >0. For SSBs and fried foods, changes were categorised as “no increase” if weekly servings increased by ≤0 or as an “increase” if servings increased by >0. Changes in physical activity levels were dichotomised into “no increase” (≤0 increased MET minutes per week) and “increase” (>0 increased MET minutes per week). Binary logistic regression analysis was conducted to assess the associations between changes in the intake of fruits, vegetables, and SSBs and changes in health knowledge scores. The model was adjusted for baseline health knowledge scores, respective baseline intake, number of sessions attended, gender, age, and race.

## 3. Results

Of the 484 individuals enrolled in the “Wise and Well” programme, 20 were excluded due to incomplete or missing surveys. The final sample comprised 464 participants (M_age_ = 73.1 years; SD = 7.6), with the majority being of Chinese descent (96.8%) and female (75.9%). Table 2 provides a detailed summary of the participants’ demographics.

Table 3 summarises the participants’ health knowledge, goal attainment, and lifestyle practices metrics before and after the programme. Significant improvements were observed in health knowledge scores (11%, *p* < 0.001), lifestyle goal attainment scaling T-scores (10 points, *p* < 0.001), and the reduction in SSBs consumption (2 servings/week, *p* = 0.002). However, there were no significant changes in fried food consumption (*p* = 0.176) or physical activity levels (*p* = 0.579). Additionally, there was a significant increase in the proportion of participants meeting or exceeding fruit and vegetable intake recommendations (fruits: 13%, *p* < 0.001; vegetables: 10%, *p* = 0.002).

Table 4 presents the outcomes of binary logistic regression analyses. Participants with “high improvement” in health knowledge had significantly higher odds of increasing their vegetable intake (adjusted OR: 2.17, 95% CI: 1.02–4.64).

## 4. Discussion

This study is among the first to evaluate the outcomes of a CBHI programme with a structured, measurable goal-setting component. It examined how the program impacted health knowledge, lifestyle goal attainment, and practices within this group. The first research hypothesis was confirmed, as health knowledge significantly improved among the participants. The second and third hypotheses were only partially supported, as the programme and improved health knowledge did not affect all lifestyle practices.

### 4.1. Effects on Health Knowledge and Lifestyle Goal Attainment

The CBHI programme significantly enhanced health knowledge and lifestyle goal attainment T-scores among older adults post-intervention. While previous research has demonstrated the effectiveness of CBHIs in improving health knowledge among older adults [40], the present study contributes new insights to the literature by integrating a structured and measurable goal-setting component (GAS) within a CBHI designed for older adults. This novel approach addresses a critical gap in existing interventions, which often lack personalised strategies to promote and sustain behaviour change among this population. A multidisciplinary team of AHPs developed and implemented the holistic intervention, which featured cultural adaptation of content, expert guidance, and a pictorial, hands-on approach tailored to older adults with varying literacy levels. These elements are widely recognised as crucial for improving health knowledge among older adults in the community [41,42]. By incorporating these aspects, the programme successfully engaged participants and enhanced their understanding of health-related information.

Although GAS has been widely used in clinical and rehabilitation settings [26,29], its application in programmes aimed at modifying lifestyle practices among community-dwelling older adults remains limited [43]. This study demonstrated that integrating GAS within a CBHI significantly improves lifestyle goal attainment. This outcome is likely driven by the goal-setting process, which enhances understanding of meaningful and challenging personal goals [44,45], boosting intrinsic motivation and active participation among older adults [46]. Furthermore, the simplicity and user-friendly design of GAS empowers participants to set realistic, measurable goals tailored to their individual needs [25], thereby promoting successful goal attainment. By establishing the effectiveness of this approach, evidence is provided for a practical strategy that addresses individual differences in needs and progress tracking, an area previously underexplored in older adults.

### 4.2. Impact on Dietary Practices and Physical Activity Levels

Significant improvements in dietary practices were also observed. Specifically, there was an increase in the proportion of participants meeting recommended dietary guidelines for fruit and vegetables and a reduction in the consumption of SSBs per week. These findings are important in Singapore, where population surveys have highlighted poor dietary practices in older adults [39]. However, while a slight decrease in fried food consumption per week was noted, it did not reach statistical significance. The widespread availability of fried food may explain this, along with the challenge of recognising items considered as fried food and the lack of viable alternatives, particularly for those relying on external food sources such as hawker centres. Nonetheless, apart from these outlier findings, the results were consistent with a recent systematic review that indicated the effectiveness of group-based interventions with behaviour change techniques in promoting healthy eating behaviours among community-dwelling older adults [47]. Future programmes could also consider providing more specific content targeting fried food sources and offering cooking classes to encourage simple, healthier meal preparation where viable alternatives are not available.

Though modest improvements in physical activity levels were observed, the change was not statistically significant. This contrasts with previous systematic reviews by Neil-Sztramko et al. and Tcymbal et al., which found that community-based physical activity interventions improved physical activity levels among older adults [47,48]. While the programme incorporated several key components known to be successful for CBHIs, such as culturally familiar activities, native language instructions, and accessibility [49], other factors may explain this outcome. Older adults may be limited by the availability or suitability of exercise programmes offered in the community. Additionally, the GPAQ does not accurately measure low intensity physical activity. Reliance on MET minutes per week as the primary measure of sufficient physical activity may not capture other benefits of the programme, such as improvements in strength and mobility. Future studies should consider using more sensitive, multidimensional measures to better evaluate the effectiveness of such interventions in older populations.

### 4.3. Associations Between Health Knowledge and Behaviour Changes

Binary logistic regression revealed the relationship between health knowledge and dietary practices, showing significant associations for some behaviours but not others. Participants with high improvements in their health knowledge had twice the odds of increasing vegetable and fruit intake; however, the odds for fruit intake were not statistically significant. This suggests that the “Wise and Well” programme’s educational content more effectively translated into increased vegetable consumption. A similar study by Hermann et al. found significant improvements in vegetable but not fruit intake post-intervention [50]. It is possible that improvements in fruit intake were inconsistent across participants or that barriers such as fruit accessibility and affordability hindered dietary changes.

High improvements in health knowledge did not lead to greater odds of reducing SSBs or fried food consumption or increasing physical activity. While knowledge is crucial for behaviour change, it is not always sufficient. Psychosocial and environmental factors can mediate the link between knowledge and behaviour change [51,52]. Self-efficacy, perceived barriers, and environmental factors likely influenced dietary and physical activity choices. Participants may have learned about the negative impacts of SSBs and fried foods but lacked confidence in preparing healthier alternatives or faced challenges like taste preferences, convenience, and the positive associations with unhealthy foods. Environmental factors such as affordability and access to healthier options also played a role. For physical activity, barriers like physical limitations, fear of injury, or lack of safe spaces for exercise may have hindered participation [53] beyond their usual regime.

### 4.4. Study Limitations

Despite its strengths, including the use of a pre-post cohort design, a large sample size, validated measures, and a targeted real-world approach, this study has some limitations that should be acknowledged. Firstly, the absence of a control group limits the ability to attribute the observed changes directly to the “Wise and Well” programme. Secondly, the sample was predominantly Chinese and female, reflecting the higher participation of Chinese women and the underrepresentation of men in community programmes in Singapore [54]. The study also excluded older adults with physical and psychological disorders, potentially introducing selection bias by omitting a subset of the population with more complex health needs. As a result, the findings cannot be generalised to the broader older adult population. Thirdly, the study focused on short-term improvements in health knowledge, goal attainment, and behaviour changes. It remains unclear whether health knowledge and goal attainment are maintained, or behaviour changes are sustained beyond the intervention period. Fourthly, the study mainly relied on subjective self-reported measures, which may introduce information partialities, such as recall bias or social desirability bias, leading participants to exaggerate or understate their lifestyle practices. Lastly, the results may have been influenced by other psychosocial and environmental factors not accounted for in the study. Future research could include a control group, recruit a more gender-balanced sample, conduct longer-term evaluations, use objective measures such as fitness trackers or perform a balance assessment where feasible, and assess other psychosocial and environmental influences.

## 5. Conclusions

The “Wise and Well” programme effectively improved health knowledge among older adults and demonstrated potential in enhancing lifestyle goal attainment. While sustained behaviour changes may require targeted interventions and more time, this study addresses a key gap in the literature by showing how a CBHI integrated with structured goal setting can deliver immediate short-term health benefits. Moreover, this scalable approach provides valuable insights for developing and implementing future CBHI programmes to promote healthy ageing in community settings, addressing a significant global challenge.

## Figures and Tables

**Table 1:** The “Wise and Well” programme outline.

Week	Session Type	Session Content
1	Group	Session 1: Lifestyle Management Theoretical foundation of behaviour change Self-assessment of daily time management Risk factor identification Lifestyle modification strategies
2	Individual	Session 2: Goal Setting and Action Planning SMART (Specific, Measurable Achievable, Relevant, Time-bound) goal methodology Review of current lifestyle practices Action plan development
3	Group	Session 3: Dietary Behaviour Modification Evidence-based nutritional guidelines Portion sizes Healthier eating choices at common local eateries
4	Individual	Session 4: Enhancing Daily Physical Activity Levels Current physical activity guidelines Intensity monitoring Safety considerations
6	Individual	Session 5: Exercise interventions for fall prevention Balance assessment protocols Evidence-based exercise prescription Environmental modification strategies
8	Group	Session 6: Stress management and time management Stress management techniques Time management strategies Cognitive behavioural approaches Social support optimisation
10	Group	Session 7: Shopping smart at the supermarket Food label interpretation Healthier choices while shopping
12	Individual	Session 8: Goal evaluation and progression Goal recalibration strategies Maintenance planning Long-term adherence strategies

**Table 2:** Demographics of study participants.

Variables	Participants *(n* = 464)
**Gender**	
Female, n (%)	352 (75.9)
**Age**	
Mean (SD)	73.1 (7.6)
Median (IQR)	72.0 (68.0–79.0)
**Race ^**	
Chinese, n (%)	448 (96.8)
Non-Chinese, n (%)	15 (3.2)
**No. of sessions attended**	
Mean (SD)	6.1 (2.0)
Median (IQR)	7.0 (5.0–8.0)

SD: standard deviation; IQR: interquartile range. ^ missing value; n = 463 race.

**Table 3:** Health knowledge, goal attainment, and lifestyle practices metrics pre- and post-programme.

Variables	Pre-Programme	Post-Programme	*p*-Value
Health Knowledge * (%)			
Mean (SD)	64.8 (21.1)	75.2 (22.9)	**<0.001**
Median (IQR)	66.7 (55.6–77.8)	80.0 (66.7–90.3)	**<0.001**
Goal Attainment * T-Score			
Mean (SD)	36.2 (11.5)	46.6 (17.8)	**<0.001**
Median (IQR)	40.0 (30.0–40.0)	50.0 (40.0–55.0)	**<0.001**
**Fruits ^ (% meeting ≥ 2 servings/day)**			
n (%)	114 (36.9)	156 (50.5)	**<0.001**
**Vegetables ^ (% meeting ≥ 2 servings/day)**			
n (%)	174 (56.3)	205 (66.0)	**0.002**
SSBs * (no. of servings/week)			
Mean (SD)	5.6 (7.9)	4.5 (6.5)	**0.002**
Median (IQR)	3.5 (0.0–7.0)	1.0 (0.0–7.0)	**0.002**
Fried Food * (no. of servings/week)			
Mean (SD)	1.1 (2.0)	0.99 (1.9)	0.176
Median (IQR)	0.3 (0–1.5)	0.2 (0.0–1.0)	0.176
**PA** **Levels ^ (% meeting** **≥600 MET/min/week)**	366 (75.6)	373 (77.1)	0.579

SD: standard deviation; IQR: interquartile range; SSBs: sugar-sweetened beverages; PA: physical activity. Results of * Wilcoxon signed-rank test and ^ McNemar’s test. Bold indicates *p* < 0.05.

**Table 4:** Association of health knowledge with healthy lifestyle practices.

Variables	Odds Ratio (95% CI)	*p*-Value
**Increase in fruits intake**		
High improvement in health knowledge	1.94 (0.94–4.03)	0.075
Low improvement in health knowledge	Reference	
**Increase in vegetable intake**		
High improvement in health knowledge	2.17 (1.02–4.64)	**0.045**
Low improvement in health knowledge	Reference	
**Decrease in SSBs intake**		
High improvement in health knowledge	1.44 (0.72–2.85)	0.301
Low improvement in health knowledge	Reference	
**Decrease** **in fried food intake**		
High improvement in health knowledge	0.70 (0.32–1.52)	0.367
Low improvement in health knowledge	Reference	
**Increase in PA levels**		
High improvement in health knowledge	0.85 (0.46–1.59)	0.615
Low improvement in health knowledge	Reference	

CI: confidence interval; SSBs: sugar-sweetened beverages. Results were adjusted for baseline health knowledge score, respective baseline intake of fruits, vegetables, SSBs, fried food, physical activity, number of sessions attended, gender, age, and race. Bold indicates *p* < 0.05.

## Data Availability

Data supporting the findings of this study are available from the corresponding author upon reasonable request.

## Supplementary Materials

The following supporting information can be downloaded at: https://www.mdpi.com/article/10.3390/healthcare12242588/s1, Health Knowledge Questionnaire.
