# Supplementary material for: Impact of a Community-Based Health Intervention on Health Knowledge, Lifestyle Goals, Dietary Practices, and Physical Activity in Older Adults: A Multisite Cohort Study
Source: Healthcare (Basel). 2024 Dec 23;12(24):2588. doi: 10.3390/healthcare12242588 (PMC11728349; doi:10.3390/healthcare12242588)
Supplement: Supplementary file 1 [file healthcare-12-02588-s001.zip › healthcare-3359977-supplementary.pdf]

Health Knowledge Questionnaire

Session 1 Lifestyle Management

1. Having a lot of rest is most important for health

   True or False

   多多休息是对健康最有益的事

   对 或 错

2. Participating in activities like mahjong and gardening is good for mental exercise

   True or False

   打麻将和园艺都是刺激脑力的活动

   对 或 错

3. It is important to set goals in order to change to a healthier lifestyle

   True or False

   想要有健康的生活，需要从设定目标开始

   对 或 错

Session 3 Dietary Behaviour Modification

1. We need to eat 1 serving of fruit and 1 serving of vegetable a day.

   True or False

   我们每天必须吃1份水果和1份蔬菜。

   对 或 错

2. According to 'My Healthy Plate', ½ of the plate is filled with rice or bread.

   True or False

   根据'我的健康餐盘', 米饭或面包占据了盘的一半。

   对 或 错

3. Added sugar from food and drinks should not exceed 10 teaspoons per day.

   True or False

   一天从食物和饮料所摄取的添加糖份不能超过10茶匙。

   对 或 错

Session 4 Enhancing Daily Physical Activity Levels

1. The Health Promotion Board (HPB) recommends 150 minutes of moderate-intensity physical activity for a week. For added health benefits, strength activities should be done on two or more days a week.

True False

保健促进局建议每周应有150分钟的中等强度的体力活动。为了增加健康益处，每周应该做两天以上的肌肉训练。

对 错

2. Engaging in regular physical activity is for the young. The elderly should lead a less active lifestyle.

True False

定期进行体力活动只适用于年轻人。老年人不必注重体力活动。

对 错

3. Warming up prior to a workout helps to prepare your body for exercise by increasing heart rate and blood circulation throughout the body.

True False

锻炼前热身有助于通过加快心率和身体的血液循环来让身体进入运动状态。

对 错

Session 5 Exercising Intervention for Falls Prevention

1. Falling is a normal process when you get older and there is nothing you can do about it.

True False

当你进入老年期，跌倒是一个正常的过程，是没有办法改变的事。

对 错

2. Falls can only result in physical injuries in the elderly.

True False

年长者跌倒只会导致身体的伤害。

对 错

3. Maintaining an active lifestyle will reduce the risk of falls.

True False

保持活跃的生活方式可以降低摔倒的风险。

对 错

Session 6 Stress Management and Time Management

1. A little bit of stress is bad for health

   True   or   False

   轻微的压力对身体有害

   对 或   错

2. Prioritizing things that are important to me is effective time management

   True   or   False

   优先处理对我重要的事情是一种有效的时间管理

   对 或   错

3. Doing light stretches will help with better sleep

   True   or   False

   做轻微的拉筋运动有助于改善睡眠

   对 或   错

Session 7 Shopping Smart at the Supermarket

1. Fruit juices that are labelled as ‘no added sugar’ do not contain sugar.

   True or False

   包装注明‘无加糖’的果汁不含糖份。

   对 或 错

2. We should use per ‘100g/mL’ on the nutritional information panel to compare food products.

   True or False

   我们应该用营养标签上的每100克/毫升的份量对比食品。

   对 或 错

3. The ‘Healthier Choice Symbol’ can help us identify healthier food products.

   True or False

   ‘较健康标签’能帮我们辨认较健康食品

   对 或 错
